# Dengue Virus Infection of Human Retinal Müller Glial Cells

**DOI:** 10.3390/v15071410

**Published:** 2023-06-21

**Authors:** Genevieve F. Oliver, Liam M. Ashander, Abby C. Dawson, Yuefang Ma, Jillian M. Carr, Keryn A. Williams, Justine R. Smith

**Affiliations:** Flinders Health and Medical Research Institute, and College of Medicine and Public Health, Flinders University, Adelaide, SA 5042, Australia; eyeota@gmail.com (G.F.O.); liam.ashander@flinders.edu.au (L.M.A.); abby.dawson@hotmail.com (A.C.D.); yuefang.ma@flinders.edu.au (Y.M.); jill.carr@flinders.edu.au (J.M.C.); keryn.williams@flinders.edu.au (K.A.W.)

**Keywords:** dengue virus, human, retina, Müller cell, retinopathy

## Abstract

Retinopathy is a recently recognized complication of dengue, affecting up to 10% of hospitalized patients. Research on the pathogenesis has focused largely on effects of dengue virus (DENV) at the blood–retinal barrier. Involvement of retinal Müller glial cells has received little attention, although this cell population contributes to the pathology of other intraocular infections. The goal of our work was to establish the susceptibility of Müller cells to infection with DENV and to identify characteristics of the cellular antiviral, inflammatory, and immunomodulatory responses to DENV infection in vitro. Primary human Müller cell isolates and the MIO-M1 human Müller cell line were infected with the laboratory-adapted Mon601 strain and DENV serotype 1 and 2 field isolates, and cell–DENV interactions were investigated by immunolabelling and quantitative real-time polymerase chain reaction. Müller cells were susceptible to DENV infection, but experiments involving primary cell isolates indicated inter-individual variation. Viral infection induced an inflammatory response (including tumour necrosis factor-α, interleukin [IL]-1β, and IL-6) and an immunomodulatory response (including programmed death-ligand [PD-L]1 and PD-L2). The type I interferon response was muted in the Müller cell line compared to primary cell isolates. The highest infectivity and cell responses were observed in the laboratory-adapted strain, and overall, infectivity and cell responses were stronger in DENV2 strains. This work demonstrates that Müller cells mount an antiviral and immune response to DENV infection, and that this response varies across cell isolates and DENV strain. The research provides a direction for future efforts to understand the role of human retinal Müller glial cells in dengue retinopathy.

## 1. Introduction

Ocular involvement in dengue virus (DENV) infection was reported as early as the 19th century [1,2,3,4,5], but only in the last few decades has retinopathy been recognized as a significant complication [6,7]. The extent to which the retina is involved was revealed after a cross-sectional study during an epidemic found a 10% prevalence in those hospitalized with dengue [8]. Dengue retinopathy is commonly based in the macular region, and the patient typically develops reduced visual acuity and visual field defects that may never completely resolve [9]. Three main forms of the condition have been reported, including cystoid macular oedema, diffuse macular oedema, and foveolitis characterized by a yellow or orange subretinal dot. Retinal electrophysiological studies have localized the pathology to the middle and outer retina [10]. Dengue retinopathy has been related temporally to the thrombocytopenia that may complicate dengue, and it has been associated with younger aged patients [8]. Other retinal manifestations are also possible, including retinal vasculitis, acute posterior multifocal placoid pigment epitheliopathy, acute zonal occult outer retinopathy, and retinochoroiditis [9].

Published studies investigating dengue retinopathy have focused largely on the cellular components of the blood–retinal barrier. This protective barrier ensures the retinal nerve tissue that is essential for vision remains isolated from circulating toxic substances and pathogens [11]. It has two components—the outer blood–retinal barrier centred on the retinal pigment epithelium and the inner blood–retinal barrier focused around the retinal vascular endothelium—both of which are maintained by a combination of tissue-specific cellular and molecular mechanisms [12]. Prior work has shown that human retinal pigment epithelial cells and retinal endothelial cells support DENV infection, and mount a type I interferon (IFN) and inflammatory response in vitro [13]. Other research has demonstrated a differential response of endothelial cell subpopulations to infection with DENV and suggested that vascular endothelial permeability may vary by cell site and viral serotype [14]. 

Müller cells are specialized glial cells unique to the retina. They are radially oriented with cell bodies located in the inner nuclear layer and processes that span the retinal thickness from the internal limiting membrane to the external limiting membrane [15]. Müller cells interact with all major anatomical compartments of the posterior segment of the eye and provide scaffolding that is critical to meticulous retinal organization [16]. Through their interaction with retinal capillaries, Müller cells form part of the inner blood–retinal barrier. They mediate neurovascular exchanges, recycle neurotransmitters, and cater to the extreme energy and oxygen requirements of inner retinal neurons [17,18]. The Müller cell has no homologue in the central nervous system, and it has been shown to perform highly specialized functions, including that of a clock that controls circadian molecular expression within the retina [19]. The role of Müller cells in dengue retinopathy is largely unknown, but there are compelling reasons why these cells warrant further study. 

Given their anatomical location, Müller cells are intimately involved with all other cell types of the neural retina. They are susceptible to infection with a range of viruses, including herpes simplex virus [20], cytomegalovirus [21], and Zika virus [22], and support infection of the apicomplexan parasite *Toxoplasma gondii* [23]. Müller cells are targets for lentiviral [24] and adeno-associated viral vectors [25], and they also interact with extracellular pathogens, such as in bacterial endophthalmitis, in which they mediate early innate immune responses through expression of Toll-like receptors and inflammatory mediators, and are capable of phagocytosis [26,27,28]. Thus, the goal of this work was to establish the susceptibility of human retinal Müller cells to infection with DENV and identify characteristics of the Müller cell response to this virus. Specifically, antiviral, inflammatory, and immunomodulatory responses after DENV infection were investigated in human Müller cells in vitro. To emulate more closely the translation to clinical disease, primary human Müller cells and field isolates of DENV were used to characterize the interactions of this unique cell with DENV.

## 2. Methods

### 2.1. Human Retinal Cell Lines

The human Müller cell line, MIO-M1 line (Moorfields Institute of Ophthalmology—Müller 1), was originally generated as spontaneously immortalized Müller cells from the retina of a 68-year-old female [29] and was generously gifted for this work by G. Astrid Limb, PhD, and Sir Peng Khaw, PhD (University College London, London, United Kingdom), under a Material Transfer Agreement (MTA-13-710). The cell line was cultured in Dulbecco’s modified Eagle’s medium (DMEM, Thermo Fisher Scientific-Gibco, Grand Island, NY, USA) and 10% foetal bovine serum (FBS, Thermo Fisher Scientific-Gibco) at 37 °C and in 5% CO_2_ in air.

The human adult retinal pigment epithelial cell line, ARPE-19 (American Type Culture Collection, cat. CRL-2302, Manassas, VA, USA), was first isolated as spontaneously immortalized retinal pigment epithelial cells from a 19-year-old male eye donor [30]. The cell line was cultured in a 1:1 DMEM:Ham’s F-12 nutrient mixture (Thermo Fisher Scientific-GIBCO) and 10% FBS (Bovogen Biologicals, Keilor East, Australia), and it was incubated at 37 °C and in 5% CO_2_ in air.

### 2.2. Primary Retinal Human Müller Cells

Primary human Müller cells were isolated from donor retinae sourced from the Eye Bank of South Australia (Adelaide, Australia) with approval of the Southern Adelaide Clinical Human Research Ethics Committee (protocol number: 175.13). A previously published protocol was followed [31], involving initial manual homogenization and subsequent digestion in 0.5 mg/mL Dispase I (Merck-Sigma Aldrich, Saint Louis, MO, USA). Cell isolates were cultured in DMEM with 1× GlutaMAX, 1× non-essential amino acids (NEAA), 1 mM sodium pyruvate, 100 U/mL penicillin-100 μg/mL streptomycin, and 10% FBS (all supplements from Thermo Fisher Scientific-Gibco), and used at passage 3 or less. 

Cell isolates were phenotyped by indirect fluorescent immunolabelling using a previously published method [23], with the following antibodies: mouse monoclonal immunoglobulin (Ig)G1 anti-human vimentin antibody (Merck-Millipore, Temecula, CA, USA, catalogue number: MAB3400, working concentration: 5 µg/mL); rabbit polyclonal IgG anti-human glutamine synthetase antibody (Merck-Sigma Aldrich, catalogue number: G2781, working concentration: 15 µg/mL); and sheep polyclonal IgG anti-human glial fibrillary acidic protein (R&D Systems, Minneapolis, MN, USA, catalogue number: AF2594, working concentration: 10 µg/mL), or a relevant negative control antibody in matched concentration, i.e., mouse monoclonal IgG1 (BD Bioscience, San Diego, CA, USA, catalogue number: 554121), or sheep or rabbit IgG (Merck-Sigma Aldrich). 

### 2.3. Dengue Virus Strains 

Six DENV field isolates were sourced from the National Environment Agency of the Environmental Health Institute of Singapore under a Material Transfer Agreement (EHI-132), representing DENV1 and DENV2 isolated from individuals hospitalized during the 2004–2005 and 2007 epidemics in Singapore (Table 1), and propagated in C6/36 mosquito cells. A seventh strain, the full-length, infectious recombinant DENV2, Mon601, was first cloned from a mouse brain-adapted New Guinea C isolate [32], and viral RNA was transfected into baby hamster kidney BKH-21 fibroblasts before amplification in C6/36 mosquito cells. 

Viral titres of Mon601 stocks were determined by plaque assay of Vero cells using neutral red overlay and were expressed in plaque-forming units (pfu)/mL [33]. The six DENV field isolates were quantified by reverse transcription (RT)–quantitative real-time polymerase chain reaction (qPCR) with reference to Mon601 pfu-equivalent units. Cell-free stocks of known pfu from Mon601-infected C6/36 cells were RNA-extracted (High Pure Viral Nucleic Acid Kit, Roche Life Sciences, Mannheim, Germany), and a Mon601 pfu:RNA ratio was calculated. Equivalent Mon601 genomes per pfu were calculated for all DENV field isolates based on RNA quantity, and viral stocks were quantified by Mon601-pfu/mL units and stored at −80 °C ahead of use in the experiments.

### 2.4. Dengue Virus Infection of Human Retinal Müller Cells

At least one day before infection, host cells were seeded for 90% confluence in either 12-well or 96-well plates. At the time of infection, frozen DENV stocks were thawed by 5 min of incubation at 37 °C. Medium was discarded from the cell monolayers, and suspensions of virus or fresh medium alone were added. Virus was applied in the minimum volume possible, but with uniform concentration across the DENV strains. Plates were incubated with rocking every 15 min to prevent drying. For infections of the MIO-M1 cell line, the viral suspension was removed after 90 min and replaced with fresh medium. For infections of the primary Müller cell isolates, the viral suspension was applied for 4 h, after which fresh medium was added to the wells without removal of the viral suspension. This variation to the more standard inoculation protocol was implemented because a pilot experiment that involved incubating one primary Müller cell isolate with Mon601 and EHI0578Y05 at a multiplicity of infection (MOI) of 5 for 90 min indicated poor infectivity of the virus by RT-qPCR of the cellular RNA extract.

Every 24 h post-inoculation (hpi), cells were checked under the microscope, and if indicated, the medium was refreshed. At the end of the experiment, cells infected in 96-well plates were fixed for immunolabelling, and cell monolayers in 12-well plates were homogenized for RNA extraction. In some infections, the culture supernatant was collected and stored at −80 °C for assay of IFN-β levels.

### 2.5. Cell Immunolabelling 

At harvest, medium was removed from the infected cell monolayers. The cells were washed with phosphate-buffered saline (PBS), fixed for 15 min with 4% paraformaldehyde, washed again with PBS, and stored at 4 °C prior to immunolabelling. Fixed cell monolayers were permeabilized for 20 min in 0.05% Triton X-100 in PBS at room temperature. Cells were then washed with PBS before blocking in PBS with 4% normal goat serum (NGS, Vector Laboratories, Burlingame, CA, USA) and 4% bovine serum albumin (Merck-Sigma Aldrich) for 30 min. The mouse J2 anti-double stranded (ds)RNA IgG2aκ (English & Scientific Consulting, Szirak, Hungary catalogue number: 10010500) and isotype-matched negative control (BD Biosciences, San Jose, CA, USA) primary antibodies diluted to 5 µg/mL in PBS with 2% NGS were applied. After overnight incubation at 4 °C, the cells were washed with PBS, and labelled with Alexa Fluor 488-tagged goat anti-mouse secondary antibody (Thermo Fisher Scientific-Molecular Probes, Eugene, OR, USA) diluted to 2 µg/mL in PBS with 2% NGS for 45 min in the dark at room temperature. Cells were then washed with PBS, fixed for 10 min with 4% paraformaldehyde, washed again with PBS, and stained with 0.1 µg/mL 4′,6-diamidino-2-phenylindole (DAPI) at room temperature. After further washing in PBS, the cell monolayers were mounted in SlowFade Gold (Thermo Fisher Scientific-Molecular Probes), and held in the dark before photographing on an IX53 microscope (Olympus Corporation, Tokyo, Japan).

### 2.6. RNA Extraction and cDNA Synthesis

RNA was extracted from infected cell monolayers with TRIzol Reagent (Thermo Fisher Scientific-Ambion, Carlsbad, CA, USA) following the manufacturer’s instructions and stored at −80 °C. The quality, purity, and concentration of the RNA were measured using a NanoDrop 2000 spectrophotometer (Thermo Fisher Scientific, Wilmington, DE, USA). cDNA was synthesized using 5X iScript Reverse Transcription Supermix (Bio-Rad, Hercules, CA, USA) to yield 20 µL of cDNA for every 250 ng of RNA template. Reverse transcription was performed in the T100 Thermocycler (Bio-Rad) under the following conditions: priming for 5 min at 25 °C; reverse transcription for 30 min at 42 °C; and inactivation for 5 min at 85 °C. cDNA was diluted to 1:10 with nuclease-free water and stored at −20 °C for later use.

### 2.7. Quantitative Real-Time Polymerase Chain Reaction

Quantitative real-time PCR was used to quantify expression of molecular transcripts. Each 20 µL PCR well contained: 2 µL of cDNA at 1:10 dilution; 1.5 µL each of forward and reverse primers at 10 µM (Table 2); 11 µL of nuclease-free water; and 4 µL of iQ SYBR Green Supermix. Plates included two replicates per sample and a ‘no template’ negative control well, replacing cDNA with 2 µL of nuclease-free water. The qPCR was performed using a Bio-Rad CFX Connect Real-Time PCR Detection System Thermocycler and CFX Connect Software (version 3.1). Amplification consisted of: a pre-cycling hold at 95 °C for 5 min; then 40 cycles of denaturation for 30 s at 95 °C, annealing for 30 s at 59 °C, and extension for 30 s at 72 °C; and a post-extension hold at 75 °C for 1 s. A melt curve was generated from a 1-s hold at 0.5 °C increments from 70 °C to 95 °C, before a hold for 5 min at 15 °C. A single fluorescence peak was produced for each primer set. The cycle threshold was measured, with the quantification cycle determination mode set to regression. Standard curves, produced with serially diluted product, confirmed PCR efficiency of 85% or greater. Size of the PCR product was confirmed by electrophoresis on a 1% agarose gel.

Viral genome copy number was determined by absolute quantification, using standard concentration curves generated from serially diluted DENV PCR product and from serially diluted PCR product of a stably expressed reference gene: glyceraldehyde-3-phosphate dehydrogense or ribosomal lateral stalk protein subunit P0. Following normalization, copy number was calculated for each sample using the starting quantity derived from the DENV PCR product standard curve. Relative gene expression was calculated for cellular samples following the method described by Pfaffl [34], using the geometric mean of two stable reference genes with CV (coefficient of variance) and M values (expression stability measurement) less than or equal to 0.25 and 0.5, respectively: ribosomal lateral stalk protein subunit P0 and β-actin. 

### 2.8. Interferon-Beta Immunoassay

The concentration of IFN-β in culture supernatants from DENV-infected cells was measured by amplified luminescent proximity homogeneous assay (Alpha)-linked immunosorbent assay (AlphaLISA). Using the ProxiPlate-384 Plus AlphaLISA kit (PerkinElmer, Waltham, MA, USA), undiluted samples were assayed in triplicates of 2 µL in each 20 µL assay, and IFN-β protein concentration in each sample was calculated by comparison with 12 human recombinant IFN-β standards, with a lower detection limit of 9.6 pg/mL.

### 2.9. Statistical Analyses

Statistical analysis was performed in GraphPad Prism software, version 7.04 (GraphPad Software, La Jolla, CA, USA). Results were described as mean ± standard deviation. Confidence intervals were calculated to demonstrate the estimated population mean. In comparative testing, statistically significant differences were defined by a *p*-value or alpha less than 0.05. Two independent conditions were compared with unpaired two-tailed Student’s *t*-test, and comparisons across multiple independent conditions were by analysis of variance (ANOVA): one-way ANOVA for one categorical independent variable and two-way ANOVA for two categorical independent variables. For longitudinal measurements, repeated measures ANOVA was used. When differences reached statistical significance, post-hoc analyses with Sidak’s test or Tukey’s multiple comparisons test were performed. The F-statistic, degrees of freedom, and exact *p*-values were calculated for each factor or interaction. 

## 3. Results

### 3.1. Susceptibility of the MIO-M1 Human Müller Cell Line to Infection with Dengue Virus

To determine the susceptibility of human Müller cells to infection with DENV, initial experiments involved infecting the spontaneously immortalized human Müller cell line, MIO-M1 [29], with Mon601, a recombinant DENV serotype 2 from the New Guinea C strain [35]. 

**Figure 1:**
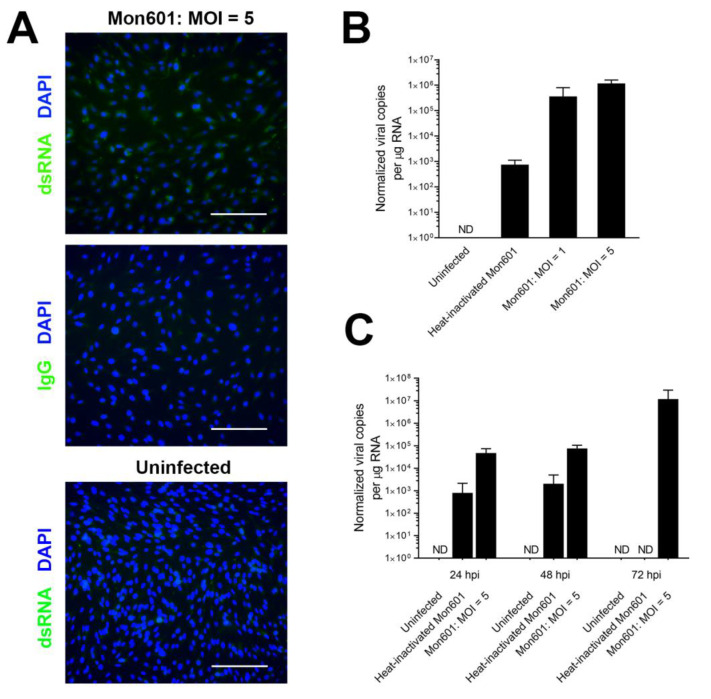
Infection of MIO-M1 human Müller cells with DENV: optimal MOI and viral load. To determine the optimal MOI for further infections, confluent monolayers of MIO-M1 cells were infected with Mon601 at MOIs of 1 and 5, mock-infected with heat-inactivated virus at an MOI of 5, or treated with medium only (n = 3 monolayers per condition). (**A**) Illustrative fluorescent images of MIO-M1 cells at 48 hpi with Mon601 at an MOI of 5 or after treatment with medium alone; and after immunolabelling to detect double-stranded RNA or isotype-matched negative control antibody. Alexa Fluor 488 (green) with DAPI (blue) nuclear counterstain. Scale bars 100 µm. (**B**,**C**). Viral RNA load of Mon601-infected MIO-M1 cells by RT-qPCR at (**B**) different viral inoculations at 48 hpi: at an MOI of 1 or 5, heat-inactivated virus at an MOI of 5, or medium only; and (**C**) different time points: 24, 48 and 72 hpi at an MOI of 5, heat-inactivated virus at an MOI of 5, or medium-only control. Genome copy number per µg RNA was normalized to reference genes (**B**) glyceraldehyde-3-phosphate dehydrogenase or (**C**) ribosomal protein lateral stalk subunit P0. Error bars denote the standard deviation of the mean. ND = no expression for at least half the replicates, n = 3–4 cultures per condition. Note logarithmic scale. Statistical comparisons were not appropriate, as some conditions yielded no product. Abbreviations: DAPI = 4′,6-diamidino-2-phenylindole, DENV = dengue virus, hpi = hours post-inoculation, MIO-M1 = Moorfields/Institute of Ophthalmology-Müller 1, MOI = multiplicity of infection, RT-qPCR = quantitative reverse transcription polymerase chain reaction.

To determine the optimal MOI for further infections, confluent monolayers of MIO-M1 cells were infected with Mon601 at MOIs of 1 and 5, mock-infected with heat-inactivated virus at an MOI of 5, or treated with medium only. At 48 hpi, cell monolayers were immunolabelled with anti-dsRNA antibody, demonstrating a productive infection of MIO-M1 cells at an MOI of 5 (Figure 1A). DENV infection was quantified by RT-qPCR as absolute viral genome copy number per µg of total RNA, using a reference gene to account for the variation in cDNA input. This procedure showed that higher viral copy numbers were present in MIO-M1 cells infected at an MOI of 5 compared to an MOI of 1 (*p* < 0.05, two-tailed *t*-test) (Figure 1B).

To characterize the kinetics of DENV infection in human Müller cells, confluent monolayers of MIO-M1 cells were infected at an MOI of 5, mock-infected with heat-inactivated virus at an MOI of 5, or left uninfected. At 24, 48, and 72 hpi, RNA was harvested. By RT-qPCR, viral RNA level was highest at 72 hpi (Figure 1C). Consistent with inactivation and degradation of virus by heat treatment, there was no increase in the number of viral genomes between 24 and 48 hpi, and no detectable viral genome at 72 hpi in the heat-inactivated DENV control condition. Imaging of cell monolayers by brightfield microscopy demonstrated a cytopathic effect (CPE) in DENV-infected cells, with increased cell debris, rounded cell bodies, and loss of cell confluence (Supplementary Figure S1). Mock-infected and uninfected Müller cells showed no CPE, indicating that inactivated virus particles were insufficient to cause macroscopic pathological changes. At 48 hpi, the CPE was obvious, but it was more profound at 72 hpi, when widespread cell death was pronounced. 

These experiments demonstrated robust infection of MIO-M1 cells with Mon601 at an MOI of 5, and 48 hpi was an optimal period to study the molecular response to infection in these cells. Given that mock infection with heat-inactivated virus generated similar results to non-infected monolayers, further infections were conducted using medium-treated cells as the negative control for infections.

### 3.2. Type I Interferon Response in Dengue Virus-Infected MIO-M1 Human Müller Cells

**Figure 2:**
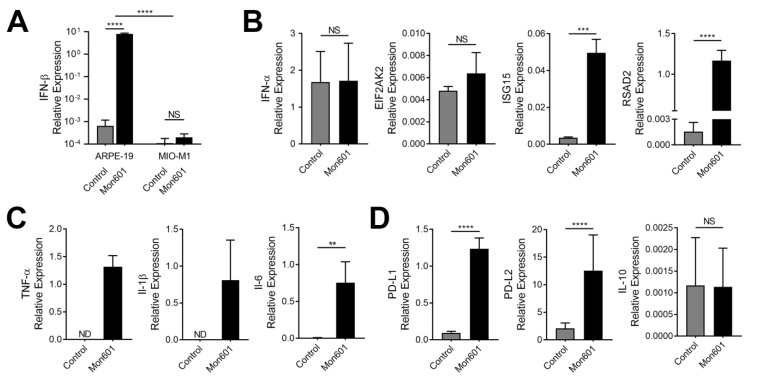
Antiviral and immune responses to DENV infection in MIO-M1 human Müller cells. To determine the antiviral and immune responses of human Müller cells to infection with DENV, the expression of key molecules was quantified by RT-qPCR. (**A**,**B**). Representative graphs from four experiments measuring expression of antiviral transcripts in Mon601-infected or medium-treated (control) cell monolayers relative to the mean of two stable reference genes, ribosomal protein lateral stalk subunit P0 (RPLP0) and β-actin. Error bars represent standard deviations of the means. (**A**). IFN-β expression in ARPE-19 and MIO-M1 cells at 48 hpi with Mon601 at an MOI of 5. Note logarithmic scale. Analysis by two-way ANOVA with Sidak’s multiple comparisons test. *** *p* < 0.0001. (**B**). Under the same conditions, cell expression of IFN-α, EIF2AK2, ISG15, and RSAD2. Analysis by unpaired two-tailed *t*-test, with n = 3–4 cultures per condition. *** *p* < 0.001, **** *p* < 0.0001. (**C**,**D**). Representative graphs from five experiments measuring the (**C**) inflammatory and (**D**) immunomodulatory response by RT-qPCR in human Müller MIO-M1 cells 48 h after inoculation with Mon601 at an MOI of 5 or medium-treated (control). Expression is displayed relative to the mean of two stable reference genes, RPLP0 and β-actin. Data were analysed by unpaired two-tailed *t*-test, with n = 3–6 cultures per condition. Error bars represent standard deviations of the mean, *** *p* < 0.001, ** *p* < 0.01. Abbreviations: ANOVA = analysis of variance, DENV = dengue virus, EIF2AK2 = eukaryotic translation initiation factor 2-alpha kinase 2; hpi = hours post-inoculation, IFN = interferon, ISG15 = interferon-stimulated gene 15, IL = interleukin, MIO-M1 = Moorfields/Institute of Ophthalmology-Müller 1, MOI = multiplicity of infection, ND = not detected in at least half the replicates, NS = not significant, PD-L = programmed death-ligand, RSAD2 = radical S-adenosyl methionine domain-containing 2; RT-qPCR = quantitative reverse transcription polymerase chain reaction.

To determine the antiviral response of human Müller cells to infection with DENV, the expression of key molecules involved in the type I IFN response was quantified by RT-qPCR. Infection of MIO-M1 cells with Mon601 did not induce expression of IFN-β transcript (Figure 2A), and this observation was replicated in three experiments, which included kinetic studies of expression at 24, 48, and 72 hpi and infections at an MOI of 1 and an MOI of 5. To determine whether this lack of response was a function of the virus and observed in other cells of the eye, another retinal cell population was infected with Mon601 using the same experimental technique, and IFN-β transcript was measured from the RNA of cell lysate by RT-qPCR. In contrast to MIO-M1 cells, the ARPE-19 human retinal pigment epithelial cell line [30] showed a robust IFN-β response by RT-qPCR compared to medium-only ARPE-19 cells (*p* < 0.0001), confirming that Mon601 can generate IFN-β in response to infection in other cells of the eye (Figure 2A). 

Further investigation was undertaken to confirm that MIO-M1 cells did not produce IFN-β in response to DENV infection. Culture supernatant was collected from MIO-M1 cells at 24 and 48 hpi with Mon601 at an MOI of 5 or medium alone. Supernatant samples were subjected to an AlphaLISA that detected IFN-β protein. No IFN-β was found in supernatant from any sample, indicating levels less than the minimum detection threshold of 9.6 pg/mL, and consistent with RT-qPCR results.

To more fully determine the type I IFN response of MIO-M1 cells in the context of DENV infection, expression of another component of the type I IFN response, IFN-α, was determined by RT-qPCR. Transcript expression of this cytokine also did not change in response to infection with DENV (Figure 2B). Similar results were obtained for the IFN-stimulated gene (ISG), eukaryotic translation initiation factor 2-alpha kinase 2 (EIF2AK2), but not ISG15 and radical S-adenosyl methionine domain-containing 2 (RSAD2), which were significantly and highly upregulated in response to infection with DENV (*p* < 0.0001, Figure 2B). Together these data suggest that, although an antiviral response to DENV is generated in infected human Müller MIO-M1 cells, this response is characterized by a lack of the key antiviral cytokine, IFN-β, and driven through IFN-β independent pathways.

### 3.3. Immune Responses of MIO-M1 Human Müller Cells to Infection with Dengue Virus

To determine the immune response of human Müller MIO-M1 cells to infection with DENV, expression of molecular transcripts involved in inflammatory and immunomodulatory processes was determined by RT-qPCR. Monolayers of MIO-M1 cells were infected with Mon601 at an MOI of 5 or mock-infected, and RNA was extracted at 48 hpi. The inflammatory response of MIO-M1 cells to infection with Mon601 was characterized by elevated expression of tumour necrosis factor (TNF)-α, interleukin (IL)-1β, and IL-6 (*p* < 0.01) compared to uninfected control cells (Figure 2C). Infected MIO-M1 cells also showed significantly increased expression of transcripts encoding two immunomodulatory cell surface molecules, programmed death-ligand (PD-L)1 (*p* < 0.0001) and PD-L2 (*p* < 0.0001), but expression of the immunomodulatory cytokine, IL-10, was unchanged following infection (Figure 2D). The immunomodulatory cell surface molecule, Fas ligand, was not detected by RT-qPCR in either infected or uninfected conditions. Together, these data indicate that Mon601-infected human Müller MIO-M1 cells mount an immune response characterized by the induction of both pro-inflammatory and immunomodulatory molecules.

### 3.4. Infection of MIO-M1 Human Müller Cells with Dengue Virus Field Isolates

To reflect human DENV infection with more fidelity and to explore the effect of the DENV strain on Müller cell response to infection, human Müller MIO-M1 cells were infected with viral isolates obtained from individuals with dengue, as well as the laboratory-adapted Mon601 recombinant strain. These DENV1 or DENV2 field isolates were collected during the 2004–2005 and 2007 epidemics in Singapore (Table 1). 

**Figure 3:**
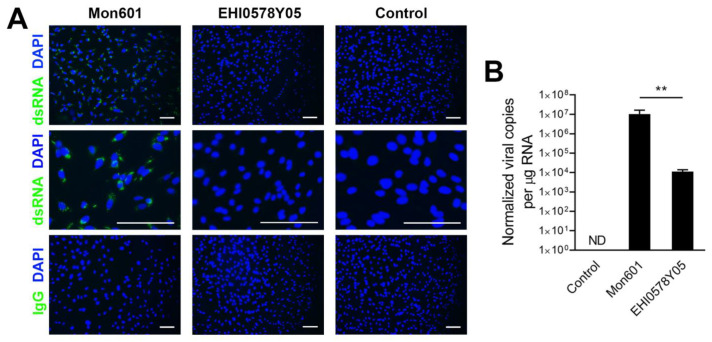
MIO-M1 human Müller cell infection with DENV field isolates. To explore the effect of DENV strain on human Müller cell responses to infection, MIO-M1 cells were infected with the laboratory-adapted Mon601 recombinant strain or one of six DENV field isolates (Table 1) at an MOI of 5, or treated with medium alone (control). Results for a representative field isolate, EHI0578Y05, are displayed. (**A**). Fluorescent photomicrographs showing cell monolayers at 48 hpi, immunolabelled for double-stranded (ds)RNA (green). Mon601-infected MIO-M1 Müller cells, but not cells infected with EHI0578Y05, stained positively for dsRNA, and no labelling was observed in uninfected cells or cells labelled with the negative control antibody. Primary antibody: J2 mouse anti-dsRNA IgG2aκ. Negative control: isotype-matched irrelevant mouse antibody. Secondary antibody: Alexa Fluor 488 goat anti-mouse IgG (green). Nuclei counterstained with DAPI (blue). All scale bars are 100 µm. (**B**). Examination of the viral RNA level of DENV-infected MIO-M1 human Müller cells and a representative field isolate, EHI0578Y05. While the field isolate was capable of infecting MIO-M1 cells, it did not replicate as efficiently as the laboratory-adapted Mon601 strain. Virus was not detected in the medium-treated control. Intracellular viral load, displayed as genome equivalents per µg RNA, normalized to reference gene, ribosomal protein lateral stalk subunit P0, n = 6 monolayers per condition. Note logarithmic scale. Error bars represent standard deviation of the mean. Analysis by two-tailed *t* test, ** *p* < 0.01. Abbreviations: DAPI = 4′,6-diamidino-2-phenylindole, hpi = hours post-inoculation, Ig = immunoglobulin, MIO-M1 = Moorfields/Institute of Ophthalmology-Müller 1, MOI = multiplicity of infection, dsRNA = double-stranded RNA.

Confluent monolayers of MIO-M1 cells were infected at an MOI of 5 with either Mon601 or one of six field isolates of DENV1 and DENV2, or treated with medium alone as negative control. At 48 hpi, monolayers were immunolabelled to detect the presence of dsRNA. The dsRNA labelling was not detected in cells infected with DENV field isolates compared to infection with Mon601 (Figure 3A). Concordant with these results, the CPE in MIO-M1 cells infected with field isolates was minimal, in contrast to the obvious CPE induced by Mon601 (Supplementary Figure S2). By RT-qPCR, which is more sensitive than immunolabelling, viral RNA was detected in cells infected with DENV field isolates; however, the copy number was significantly lower for cells infected with DENV field isolates in comparison with Mon601 (Figure 3B). A medium-treated control was included, from which no viral copies were detected. These data indicate that, although the DENV field isolates are capable of infecting MIO-M1 cells, they do not replicate as well as Mon601 in these cells.

**Figure 4:**
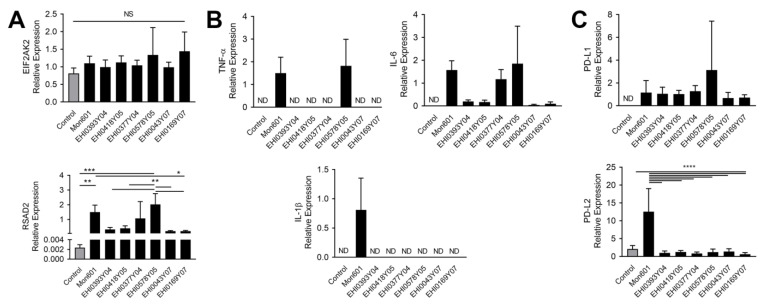
Molecular responses of MIO-M1 human Müller cells infected with DENV field isolates. Human Müller MIO-M1 cells were infected with Mon601 or each of six DENV field isolates at an MOI of 5 and harvested at 48 hpi. Transcript levels of seven mediators (EIF2AK2, RSAD2, TNF-α, IL-1β, IL-6, PD-L1, PD-L2) were measured by RT-qPCR. Expression is displayed relative to the mean of two stable reference genes, ribosomal protein lateral stalk subunit P0 and β-actin, n = 6 monolayers per condition. Error bars show the standard deviation of the mean. Differential expression of mediators was seen across infection with DENV1 (EHI0393Y04, EHI0418Y05, EHI0043Y07, EHI0169Y07) and DENV2 (Mon601, EHI0377Y04, EHI0578Y05) strains. (**A**) Anti-viral transcripts, EIF2AK2 and RSAD2; (**B**) inflammatory transcripts, TNF-α, IL-1β, and IL-6; and (**C**) immunomodulatory transcripts, PD-L1 and PD-L2. For transcripts present across all conditions, data were analysed by one-way ANOVA with Tukey’s post-hoc multiple comparisons test, * *p* < 0.05, ** *p* < 0.01, *** *p* < 0.001, **** *p* < 0.0001. Abbreviations: ANOVA = analysis of variance, DENV = dengue virus, EIF2AK2 = eukaryotic translation initiation factor 2-alpha kinase 2, hpi = hours post-inoculation, IL = interleukin, MIO-M1 = Moorfields/Institute of Ophthalmology-Müller 1, MOI = multiplicity of infection, ND = expression not detected in ≥ 4 of 6 replicates, NS = not significant, PD-L = programmed death-ligand, RSAD2 = radical S-adenosyl methionine domain-containing 2, RT-qPCR = quantitative reverse transcription polymerase chain reaction, TNF-α = tumour necrosis factor-alpha.

To understand the antiviral and immune responses of human Müller cells to infection with the DENV field isolates, transcripts were measured by RT-qPCR. Monolayers of human Müller MIO-M1 cells were infected at an MOI of 5 with each one of six DENV field isolates, infected with Mon601, or left uninfected. At 48 hpi, IFN-β was not detected in any samples, indicating an absence of expression of the antiviral cytokine, both in the unstimulated state and in response to DENV infection (not shown). Expression of EIF2AK2 was not altered at 48 hpi, but expression of RSAD2 was increased significantly in all DENV-infected cells, with higher expression in DENV2 strain-infected monolayers (Mon601, EHI0377Y04 and EHI0578Y05, *p* < 0.0001) (Figure 4A). 

Transcripts encoding the inflammatory cytokines, TNF-α, IL-1β and IL-6, were not consistently detected in monolayers subjected to mock infection with medium alone, but infection with all of the DENV isolates stimulated IL-6 expression, although notably higher levels were seen with DENV2 strains (Figure 4B). Infection with Mon601 induced production of TNF-α and IL-1β RNA, and the EHI0578Y05 strain also stimulated expression of TNF-α RNA. Expression of the immunomodulatory transcript, PD-L1, was upregulated with infection with all DENV strains, and PD-L2 expression was relatively high in Mon601-infected cells (*p* < 0.001, Figure 4C). The results show that differential molecular responses of human Müller MIO-M1 are seen across infection with DENV1 and DENV2 strains. The DENV2 isolate EHI0578Y05, was used for further analysis since it showed the best replication and induction of immune responses.

### 3.5. Responses to Dengue Virus Infection in Primary Human Müller Cells

**Figure 5:**
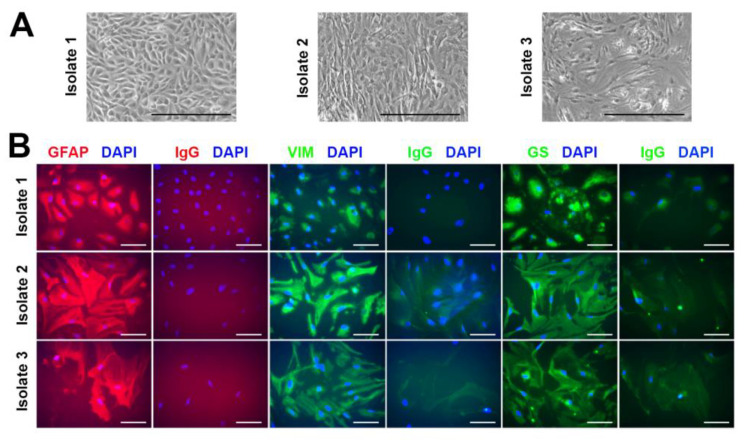
Expression of cell markers in primary human Müller cell isolates. (**A**). Photomicrographs of primary human Müller cell monolayers isolated from eyes of three male cadaveric donors. Scale bars 100 µm. (**B**). Fluorescent photomicrographs of human Müller cell monolayers immunolabelled for Müller cell markers: intermediate filaments glial fibrillary acidic protein (GFAP, red), vimentin (VIM, green), or glutamine synthetase (GS, green). Negative controls were labelled with species-matched polyclonal or monoclonal antibodies targeted to irrelevant antigens. Primary antibodies: sheep anti-GFAP IgG, mouse anti-VIM IgG1K, rabbit anti-GS IgG. Secondary antibodies: Alexa Fluor 594 donkey anti-sheep IgG (red), Alexa Fluor 488 goat anti-mouse (green), Alexa Fluor 488 goat anti-rabbit (green). Nuclei counterstained with DAPI (blue). Scale bars 50 µm. Abbreviations: DAPI = 4′,6-diamidino-2-phenylindole, GFAP = glial fibrillary acidic protein, GS = glutamine synthetase, Ig = immunoglobulin, VIM = vimentin.

To further approximate in vivo responses of human Müller cells to DENV infection, primary human Müller cells were used to investigate molecular responses to infection with the six DENV field isolates (Table 1), as well as recombinant DENV2 Mon601. Primary human Müller cell isolates were prepared from eyes of three male cadaveric donors aged 68 to 82 years old, obtained within a mean of 26 h post-mortem. To characterize these cell isolates prior to use in experiments, the cells were immunolabelled for markers of cultured primary human Müller cells: glutamine synthetase, vimentin, and glial fibrillary acidic protein. All cell isolates stained positively for these markers, in comparison with cells stained with negative control antibodies, consistent with a Müller cell phenotype (Figure 5).

**Figure 6:**
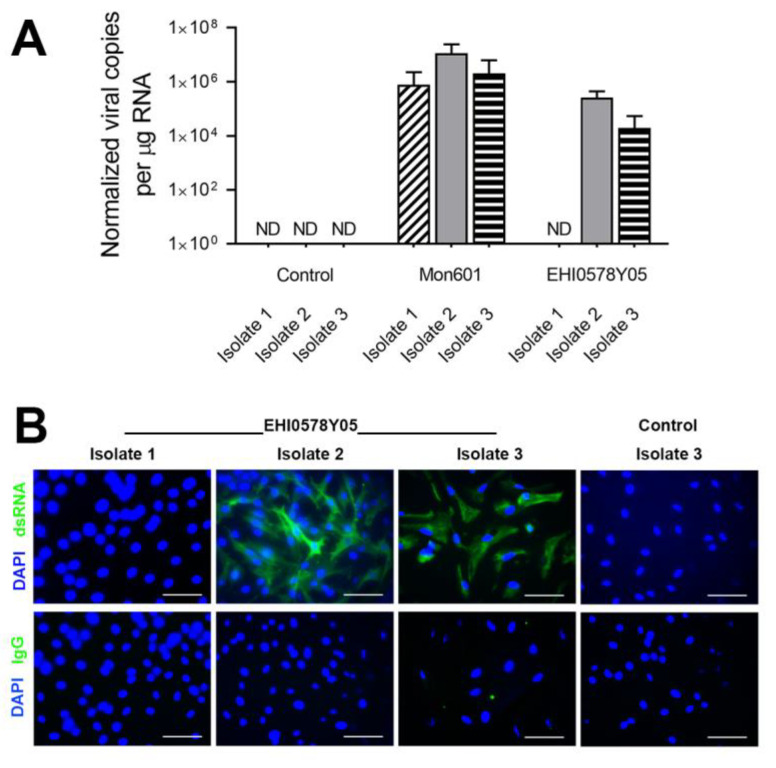
Infection of primary human Müller cell infection with DENV. Monolayers of primary human Müller cells were infected with Mon601 or the representative field strain, EHI0578Y05. (**A**). Quantification of viral genomes in primary Müller cell isolates by RT-qPCR at 48 hpi with Mon601 or EHI0578Y05 at an MOI of 5, or control cells treated with medium alone. Expression normalized to reference gene, ribosomal protein lateral stalk subunit P0, n = 4 monolayers per condition. Error bars show standard deviation of the mean. Note logarithmic scale. Analysis was by two-tailed unpaired *t* test; viral load was significantly greater in Mon601 compared with EHI0578Y05, *p* < 0.01. (**B**). Fluorescent photomicrographs showing immunolabelling of double-stranded (ds)RNA (green) in primary human Müller isolates at 48 hpi with representative DENV field strain, EHI0578Y05, at an MOI of 5. Nuclei counterstained with DAPI (blue). Müller isolates 2 and 3 show obvious presence of dsRNA in DENV-inoculated cells. Primary antibody: J2 mouse anti-dsRNA IgG2aκ, negative control: isotype-matched irrelevant mouse antibody. Secondary antibody: Alexa Fluor 488 goat anti-mouse (green). Scale bars 100 µm. Abbreviations: DAPI = 4′,6-diamidino-2-phenylindole, DENV = dengue virus, hpi = hours post-inoculation, Ig = immunoglobulin, MIO-M1 = Moorfields/Institute of Ophthalmology-Müller 1, MOI = multiplicity of infection, ND = expression not detected in ≥ 3 of 4 replicates, dsRNA = double-stranded ribonucleic acid, RT-qPCR = quantitative reverse transcription polymerase chain reaction.

Monolayers of primary human Müller cells were infected with Mon601 or a representative field strain, EHI0578Y05, at an MOI of 5. At 48 hpi, the CPE varied across Müller isolates and DENV strains, but was most apparent for Müller cell isolate 3 following infection by Mon601 (Supplementary Figure S3). At this point, RNA was extracted from cells, or monolayers were fixed for immunolabelling. Viral RNA load, as measured by RT-qPCR, reached higher levels in cell isolates infected with Mon601 compared with EHI0578Y05 (Figure 6A). Immunolabelling of dsRNA demonstrated greater infection of Müller isolate 2, with nearly all cells appearing infected (Figure 6B), consistent with the results obtained by RT-qPCR.

To investigate the molecular responses of the three primary human Müller cell isolates to DENV infection, analysis of transcript expression at 48 hpi was measured by RT-qPCR of extracted RNA. Experimental conditions comprised control cells treated with medium alone, and cells infected with Mon601 or EHI0578Y05 field strain, both at an MOI of 5.

**Figure 7:**
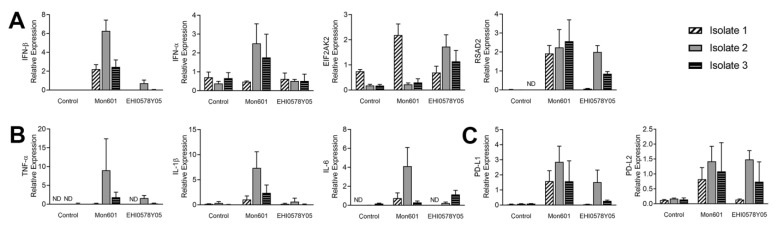
Responses of primary human Müller cell isolates to infection with DENV. To investigate the molecular responses of the primary human Müller cell isolates to DENV infection, transcript expression of nine mediators, representing antiviral (**A**), inflammatory (**B**), and immunomodulatory (**C**) responses, was measured by RT-qPCR at 48 hpi in each of three Müller cell isolates infected with Mon601 or the representative field isolate, EHI0578Y05, at an MOI of 5, or not infected, n = 4 cultures per condition. Expression is displayed relative to the mean of two stable reference genes, ribosomal protein lateral stalk subunit P0 (RPLP0) and β-actin. Error bars represent standard deviations of the means. Statistical analysis was performed by two-way ANOVA with Turkey’s post-hoc multiple comparisons test (see Supplementary Tables S1 and S2), where expression was greater than zero (i.e., not performed for RSAD2, TNF-α or IL-6). Expression of components of the type I IFN pathway showed significant variation across cells, including IFN-α (df 4, mean squares 1.707, F (4, 25) = 4.767, *p* < 0.01) and EIF2AK2 (df 4, mean squares 2.487, F (4, 27) = 31.39, *p* < 0.0001). IL-1β transcript expression was significant for the interaction of infection status with primary Müller isolate (df 4, mean squares 12.51, F (4, 25) = 7.490, *p* < 0.001), and infection with Mon601 most strongly influenced IL-1β expression. Infection with DENV resulted in increased expression of both PD-L1 (df 2, mean squares 11.94, F (2, 27) = 26.36, *p* < 0.0001) and PD-L2 (df 2, mean squares 2.895, F (2, 27) = 13.87, *p* < 0.0001), and PD-L1 expression was higher with Mon601 compared to EHI0578Y05. The influence of primary Müller isolate on transcript expression was smaller, with *p* < 0.01 for both PD-L1 (df 2, mean squares 3.117, F (2, 27) = 6.880) and PD-L2 (df 2, mean squares 1.341, F (2, 27) = 6.427), indicating that expression of immunomodulatory transcripts, PD-L1 and PD-L2, was influenced more by DENV infection than by individual cell isolates. Abbreviations: ANOVA = analysis of variance, DENV = dengue virus, EIF2AK2 = eukaryotic translation initiation factor 2-alpha kinase 2, hpi = hours post-inoculation, IFN = interferon, IL = interleukin, MOI = multiplicity of infection, ND = expression not detected in ≥ 3 replicates, PD-L = programmed death-ligand, RSAD2 = radical S-adenosyl methionine domain-containing 2, RT-qPCR = quantitative reverse transcription polymerase chain reaction, TNF-α = tumour necrosis factor-alpha.

First, the antiviral type I IFN response of Müller isolates to DENV infection was investigated (Figure 7A). In contrast to the lack of IFN-β induction in DENV-infected MIO-M1 cells, primary Müller cells expressed IFN-β mRNA in response to DENV infection, but the magnitude varied between Müller cell isolates. Expression of other components of the type I IFN pathway showed significant variation across cells and by virus strain, and these molecules included IFN-α (*p* < 0.01) and EIF2AK2 (*p* < 0.0001). Interestingly, expression of EIF2AK2 was higher for EHI0578Y05 compared to Mon601 infection in two of three Müller cell isolates (isolates 2 and 3), whereas expression was lower for EHI0578Y05 versus Mon601 infection in the third Müller cell isolate (isolate 1). The transcript RSAD2 had very low constitutive expression that was not detected in one Müller cell isolate, and higher induction in response to Mon601 than EHI0578Y05 infection. Because expression was not detected in one condition, statistical analysis could not be performed for this transcript. 

Second, the inflammatory response of primary human Müller cells to infection with DENV was explored (Figure 7B). In two of three primary cell isolates, TNF-α was not expressed at baseline, but in one isolate, strong induction was seen following Mon601 infection and a modest induction after EHI0578Y05 infection. This same pattern of expression was observed for IL-6. Analysis of IL-1β transcript expression demonstrated significance for the interaction of infection status with primary Müller isolate (*p* < 0.001), and infection with Mon601 was the factor that most strongly influenced IL-1β expression. 

Finally, the immunomodulatory response to DENV infection was explored by measuring expression of PD-L1 and PD-L2 transcripts (Figure 7C). Infection with DENV resulted in increased expression of both PD-L1 (*p* < 0.0001) and PD-L2 (*p* < 0.0001), and PD-L1 expression was higher with Mon601 in comparison to EHI0578Y05. The influence of the primary Müller isolate on transcript expression was smaller, with *p* < 0.01 for both PD-L1 and PD-L2. These results showed that expression of these immunomodulatory transcripts was influenced more by DENV infection than by individual cell isolates, and that patterns of increased expression in response to infection differed across donors. 

In summary, in contrast to the MIO-M1 human Müller cell line, primary human Müller cells mount a type I IFN response to infection with DENV, and this response is characterized by expression of IFN-β, IFN-α, and ISGs. Inflammatory and immunomodulatory responses tended to be higher with Mon601 than with EHI0578Y05 infection.

## 4. Discussion

This work demonstrates that human Müller cells are susceptible to DENV infection, and provides a description of the Müller cell response to infection with DENV1 and DENV2 isolates. Initially infection was tested in human Müller MIO-M1 cells with the laboratory-adapted strain Mon601, and productive infection was demonstrated. The infection was characterized by a limited antiviral response that lacked components of the type I IFN pathway, including IFN-β, the key type I IFN. Immune responses generated with DENV infection included upregulation of the pro-inflammatory cytokines, TNF-α, IL-1β, and IL-6, which have also been documented in studies of Müller cells exposed to other infectious pathogens [27]. An immunomodulatory response to DENV infection was also seen, with increased expression of PD-L1 and PD-L2.

Follow-up experiments used DENV1 and DENV2 strains isolated from the field to infect the MIO-M1 cells. Infection with these strains was relatively poor compared to Mon601 infection, but differential responses were seen across the strains, with greater antiviral, inflammatory, and immunomodulatory changes following exposure to DENV2 strains compared to DENV1. Primary human Müller cells isolated from cadaveric donor eyes were also infected with Mon601 and one representative field strain. In contrast to the MIO-M1 cell line, primary Müller cells expressed very low levels of IFN-β constitutively, and this antiviral cytokine was upregulated in the context of DENV infection. Other molecular components of the type I IFN response were also upregulated after DENV infection, namely IFN-α and ISGs, EIF2AK2, ISG15, and RSAD2, and these responses showed variation across individual cell isolates. These results suggest that in vivo, human retinal Müller cells are susceptible to infection and mount inflammatory and antiviral responses to DENV.

Pathological examinations of eyes with dengue retinopathy have not been described, and there have been no reports of DENV isolated from the ocular fluid of patients. In a case series of epidemic retinitis, aqueous humour in one patient with dengue was tested for DENV by RT-qPCR, and this test returned a negative result [36]. In another series of posterior uveitis cases in Singapore, investigators found that DENV was the leading cause of infectious retinitis during dengue epidemics, and although the authors noted that PCR testing of ocular fluid was performed in cases of presumed infectious uveitis, they did not state whether there was specific testing for DENV [37]. Positive confirmation of DENV in ocular tissue was presented in a case report that described isolation of DENV3 from a corneal donor who died of a febrile illness. Virus was identified by RT-qPCR and plaque assay of corneal tissue suspension [38]. Thus, there is no histopathological or laboratory confirmation of direct DENV infection of the human retina in clinical disease. In severe dengue, virus has been isolated from cerebrospinal fluid [39], and post-mortem studies have demonstrated DENV RNA, antigen, and IgM antibodies in the brains of individuals with meningitis and encephalitis, indicating that the virus had crossed the blood–brain barrier [40,41,42,43]. In a mouse model of systemic DENV infection, inflammatory responses were observed in the eyes of infected AG129 mice [44]. Thus, it is quite plausible that DENV crosses the blood–retinal barrier and infects Müller cells. 

The type I IFN response is the key human antiviral response, seen in DENV-infected human cells in vitro, as well as in clinical dengue [45,46,47]. This work demonstrated that the antiviral response to DENV in primary, but not MIO-M1, human Müller cells is characterized by expression of its primary driver, IFN-β [48]. This cytokine acts in an autocrine and paracrine fashion by engaging IFN α/β receptors on the cell surface, ultimately activating IFN regulatory factor 3, a transcription factor that drives expression of ISGs, including RSAD2, EIF2AK2, and ISG15 [49]. Thus, infected Müller cells expressing IFN-β would also prime neighbouring retinal cell populations to switch to an antiviral state, and the Müller cells’ strategic location at the inner blood–retinal barrier makes them one of the cell populations likely to be infected in the early stages of infection with vascular-borne pathogens. The cytokine, IFN-α, plays an overlapping and synergistic role in the type I IFN response and binds to the same IFN α/β receptors [50]. In this work, IFN-α was upregulated in primary Müller cells in response to DENV infection.

Despite their name, some ISGs are also expressed independently of IFN-α and IFN-β [51,52]. This finding was demonstrated in the current work involving DENV-infected MIO-M1 cells. The IFN-β protein was not detected by AlphaLISA in infected or uninfected MIO-M1 cells, and transcript expression of IFN-α or EIF2AK2 was not altered with DENV infection. In contrast, expression of RSAD2 and ISG15 transcripts was increased. Similar observations of the MIO-M1 cell response to infection were reported in a study focused on Zika virus infection [53]. Expression of these antiviral molecules through type I IFN-independent signalling pathways has been observed in viral infections [45,54,55,56,57,58,59,60]. The ISG15 molecule is an intrinsically multifunctional protein involved in diverse cell processes, and it plays a key role in the cellular innate immune response, acting as an extracellular cytokine, as well as enhancing intracellular antiviral pathways and binding to viral proteins [57]. Another broad-spectrum molecule, RSAD2—known also as viperin or virus inhibitory protein, endoplasmic reticulum-associated, IFN-inducible—is strongly expressed in response to diverse viral infections and inhibits early viral replication of DENV [61] and other flaviviruses [56,62]. 

Alterations to the expression of inflammatory molecules were observed in response to DENV infection of Müller cells. Increased expression of TNF-α, IL-1β, and IL-6 was seen predominantly with DENV2 infection and at higher levels with Mon601 compared with DENV1 and DENV2 field isolates. Inflammation is a characteristic of murine infection with DENV [44], and high levels of circulating TNF-α and IL-1β in patients are associated with severe dengue [63,64]. Retinopathy associated with DENV often presents with signs of posterior-segment inflammation, and the spectrum of disease includes vasculitis and retinitis [65]. Macular oedema, a prominent feature of dengue retinopathy [6,8,10,66,67,68,69,70,71,72,73,74,75], represents breakdown of the BRB [76]. Müller cells perform an important role in controlling retinal fluid homeostasis [16], and direct infection of the cells could impair their function in this critical process, leading to excess fluid in the retinal interstitium with consequent macular oedema. 

This work revealed molecular adjustments of components of immunomodulatory pathways. DENV-infected Müller cells showed increased expression of programmed death-ligands PD-L1 and PD-L2. These ligands interact with programmed cell death protein 1 (PD-1), which is expressed on activated T-cells and other immune cells, to form an inhibitory signal that prevents an excessive immune response by blocking production of pro-inflammatory cytokines [77]. The PD-1/PD-L1 pathway also mediates immune responses to acute viral infection through suppression of T-cell-mediated tissue damage [78]. Upregulation of PD-L1 has been seen in acute and chronic viral infections [79]. Dendritic cells infected with DENV2 showed increased PD-L2 and decreased PD-L1 expression, expected to preferentially inhibit T-helper 1 effector cell responses [80]. While expression of both PD-L1 and PD-L2 was induced with DENV infection in this work, relatively high expression of PD-L2 was seen in MIO-M1 cells infected with DENV2 (Mon601). This finding was not reproduced in primary Müller cell infections and may be a function of the unique MIO-M1 transcriptome [81]. Müller cells may participate in ocular immune privilege through the expression of immunomodulatory genes, PD-L1 and PD-L2, and other ocular cell populations have also shown expression of these ligands [82].

Previous work has concentrated on retinal pigment epithelial cells and retinal endothelial cells and their participation in blood–retinal barrier changes in DENV infection. One report detailed responses of primary human retinal pigment epithelial cells, ARPE-19 cells, and human retinal endothelial cells infected with two strains of DENV2, and showed that infected endothelial cells increased their expression of cell adhesion molecules [13]. Other investigators studied responses across different endothelial cell subpopulations and four DENV serotypes, including commercially sourced primary human retinal endothelial cells. Researchers demonstrated loss of barrier function in retinal endothelial cells over time that was DENV serotype-dependent [14]. Across all serotypes, DENV infection induced a reduction in endothelial cell membrane permeability that was greatest with DENV1 and DENV2, and a qualitative change in permeability in the early stage of infection was seen with DENV1. Effects on junctional molecules were not evaluated, but permeability changes were linked to the loss of cell–cell interactions, as well as their effect on cell membrane capacitance. An investigation of human retinal pigment epithelial cell infections with DENV1 and DENV2 strains revealed that viral strain, as well as serotype, influenced the cellular responses [83].

Significant differences between primary isolates and cell lines are becoming apparent. Recent transcriptomic analysis of human retinal cell populations elegantly demonstrated that the MIO-M1 cell line forms a distinct cluster that bears more similarity to retinal astrocytes than to primary human Müller cells [81]. A curious finding in this research was the lack of a type I IFN response in DENV-infected MIO-M1 cells. However, validation of DENV infection in isolated primary human Müller cells confirmed that primary Müller cells do generate both IFN-β and IFN-α in response to infection with DENV. This finding underscores the importance of using isolated human primary cells in research, as cell lines may generate idiosyncratic results that do not reflect in vivo biology. The primary human Müller cells in this work displayed variation in terms of their cell phenotype and molecular responses to DENV infection, and biological and technical factors are likely to have contributed to these differences. Individuals differ in their responses to biological conditions, and this natural variation is also to be expected in cell culture. 

## 5. Conclusions

This work has demonstrated that human Müller cells mount an antiviral and immune response to DENV infection characterized by expression of pro-inflammatory cytokines, immunomodulatory molecules, and the type I IFN response, and that this response varies across cell isolates and DENV strains. This research provides a foundation and direction for future research efforts that will help us to understand the pathogenesis of dengue retinopathy.

## Figures and Tables

**Table 1:** Dengue virus serotype 1 and 2 field isolates collected during dengue epidemics in Singapore.

Strain Name	GenBank ID	Serotype	Year of Isolation
EHI0393Y04	EU069606.1	DENV1	2004
EHI0418Y05	EU069594.1	DENV1	2005
EHI0377Y04	JN851123.1	DENV2	2004
EHI0578Y05	JN851126.1	DENV2	2005
EHI0043Y07	GQ357691.1	DENV1	2007
EHI0169Y07	GQ357690.1	DENV1	2007

**Table 2:** Primer sequences and expected product sizes for gene transcripts.

Transcript		Primer Sequences	Expected Product Size (bp)
DENV	FWD REV	5′-CTA TGC TAA GCT TGA GCC CCG TC-3′ 5′-CGG ATC CTC TAG AAC CTG TTG-3′	400
IFN-α	FWD REV	5′-CAA GGT TCA GAG TCA CCC ATC-3′ 5′-CAG AGA GCA GCT TGA CTT GCA-3′	120
IFN-β	FWD REV	5′-AAA CTC ATG AGC AGT CTG CA-3′ 5′-AGG AGA TCT TCA GTT TCG GAG G-3′	168
ISG15	FWD REV	5′-GAG AGG CAG CGA ACT CAT CT-3′ 5′-AGC ATC TTC ACC GTC AGG TC-3′	231
RSAD2	FWD REV	5′-TGA CGG AAC AGA TCA AAG CA-3′ 5′-GCA CCA AGC AGG ACA CTT CT-3′	174
TNF-α	FWD REV	5′-TCT CGA ACC CCG AGT GAC AA-3′ 5′-TGA AGA GGA CCT GGG AGT AG-3′	482
IL-1β	FWD REV	5′-TGA CCT GAG CAC CTT CTT TC-3′ 5′-CAG CTG TAG AGT GGG CTT ATC-3′	331
IL-6	FWD REV	5′-ATG AAC TCC TTC TCC ACA AGC GC-3′ 5′-GAA GAG CCC TCA GGC TGG ACT G-3′	628
IL-10	FWD REV	5′-GTG ATG CCC CAA GCT GAG A-3′ 5′-GCA TCT GGC AAC CCT ACA ACA AG-3′	138
PD-L1	FWD REV	5′-ACG CAT TTA CTG TCA CGG TTC C-3′ 5′-GAC TTC GGC CTT GGG GTA GC-3′	446
PD-L2	FWD REV	5′-CAA CTT GGC TGC TTC ACA TTT T-3′ 5′-TGT GGT GAC AGG TCT TTT TGT TGT-3′	137
FASL	FWD REV	5′-GGG TGG ACT GGG GTG GCC TAT-3′ 5′-GGA TTG GGC CTG GGG ATG TTT CA-3′	126
RPLP0	FWD REV	5′-GCA GCA TCT ACA ACC CTG AA-3′ 5′-GCA GAT GGA TCA GCC AAG AA-3′	235
β-Actin	FWD REV	5′-TCA AGA TCA TTG CTC CTC CTG AG-3′ 5′-ACA TCT GCT GGA AGG TGG ACA-3′	87
GAPDH	FWD REV	5′-AGC TGA ACG GGA AGC TCA CTG G-3′ 5′-GGA GTG GGT GTC GCT GTT GAA GTC-3′	209

Abbreviations: bp = base pairs, DENV = dengue virus, EIF2AK2 = eukaryotic translation initiation factor 2-alpha kinase 2, FWD = forward, FASL = Fas ligand, GAPDH = glyceraldehyde-3-phosphate dehydrogenase, IFN = interferon, IL = interleukin, ISG = interferon-stimulated gene, PD-L = programmed death-ligand, REV = reverse, RPLP0 = ribosomal protein lateral stalk subunit P0, RSAD2 = radical S-adenosyl methionine domain-containing 2, TNF-α = tumour necrosis factor-alpha.

## Data Availability

Data are contained within the article or Supplementary Materials.

## Supplementary Materials

The following supporting information can be downloaded at: https://www.mdpi.com/article/10.3390/v15071410/s1, Figure S1: Infection of MIO-M1 human Müller cells with DENV Mon601. Figure S2: Infection of MIO-M1 human Müller cells with DENV field isolates. Figure S3: Infection of primary human Müller cells with DENV. Table S1: Results of statistical analyses by two-way ANOVA for Figure 7. Table S2: Results of statistical analyses by Tukey’s multiple comparisons test for Figure 7.
