# Supplementary material for: Dengue Virus Infection of Human Retinal Müller Glial Cells
Source: Viruses. 2023 Jun 21;15(7):1410. doi: 10.3390/v15071410 (PMC10385653; doi:10.3390/v15071410)
Supplement: Supplementary file 1 [file viruses-15-01410-s001.zip › viruses-2445251-supplementary.pdf]

SUPPLEMENTARY MATERIAL

Figure S1. Infection of MIO-M1 human Müller cells with DENV Mon601. Representative brightfield photomicrographs of Mon601-infected MIO-M1 cells at a multiplicity of infection (MOI) of 5, and medium-treated (control) MIO-M1 cells. MIO-M1 cells inoculated with heat-inactivated virus had a similar appearance to cells treated with medium alone (not shown). Evaluated time points = 24, 48, 72 hours post-inoculation (hpi). Scale bars = 100 µm.

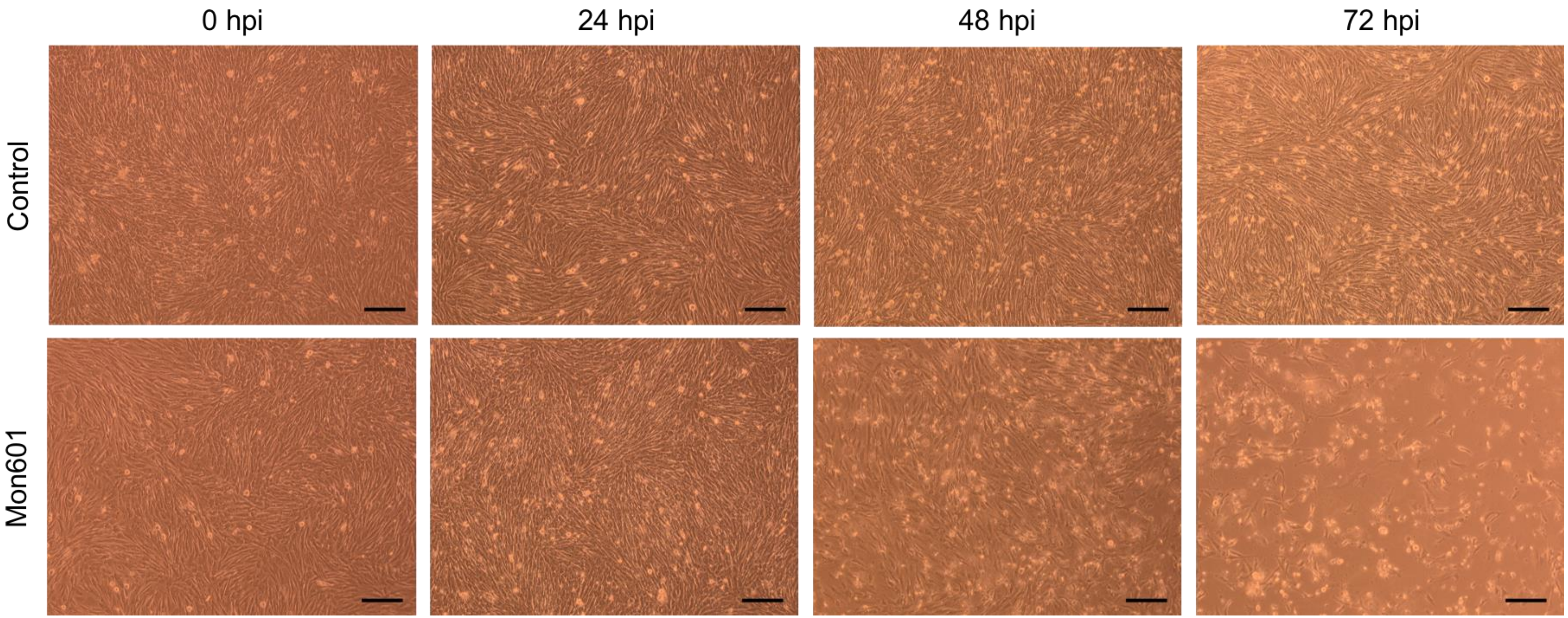

Figure S2. Infection of MIO-M1 human Müller cells with DENV field isolates. MIO-M1 cells were infected with the laboratory-adapted Mon601 recombinant strain or one of 6 DENV field isolates (Table 1) at an MOI of 5, or treated with medium alone (control). Representative brightfield photomicrographs demonstrating cytopathic effect (CPE) at 48 hpi for Mon601-infected, representative field strain EHI0578Y05-infected, and control cells. Scale bars = 100 µm.

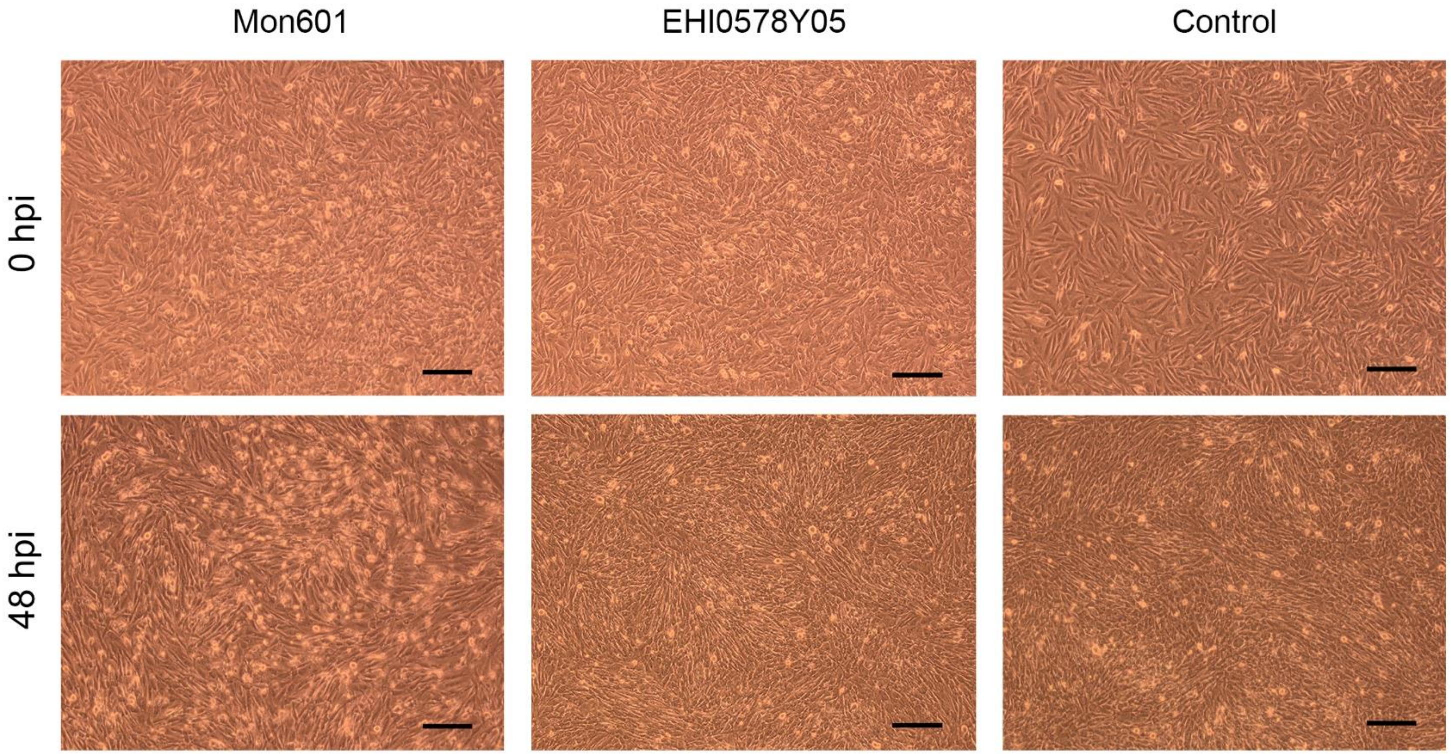

Figure S3. Infection of primary human Müller cells with DENV. Monolayers of primary human Müller cells were infected with Mon601 or the representative field strain, EHI0578Y05, at an MOI of 5, or treated with medium alone (control). Representative brightfield images demonstrating CPE at 48 hpi. Scale bars = 100 μm.

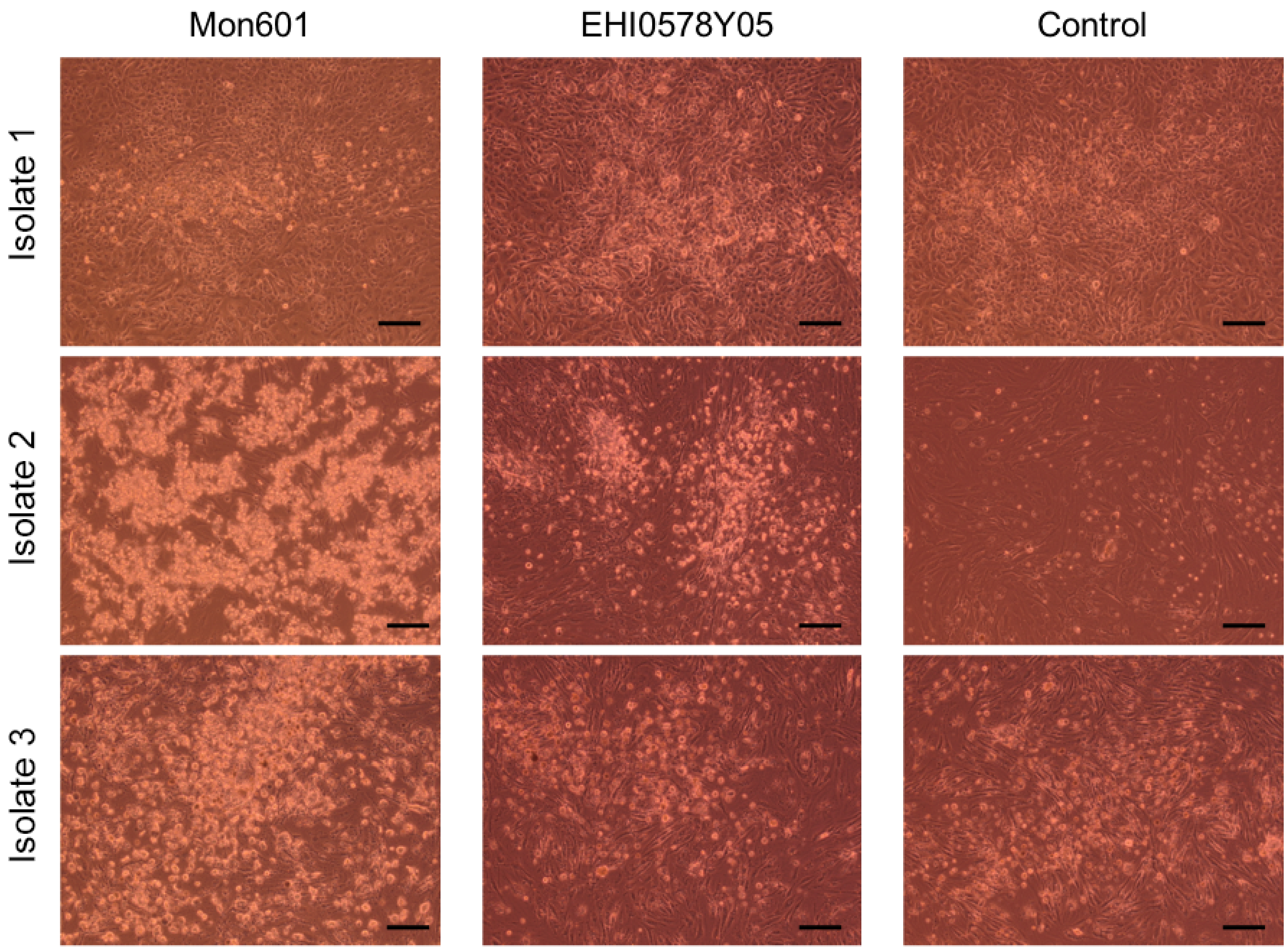

Table S1. Results of statistical analyses by two-way ANOVA for Figure 7.

Transcript	Source of variation	df	Mean squares	F ratio	P-value	
IFN-β	Interaction	4	5.903	F (4, 27) = 23.98	<0.0001	****
	Infection status	2	49.81	F (2, 27) = 202.3	<0.0001	****
	Cell population	2	9.626	F (2, 27) = 39.10	<0.0001	****
IFN-α	Interaction	4	1.707	F (4, 25) = 4.767	0.0054	**
	Infection status	2	3.971	F (2, 25) = 11.09	0.0004	***
	Cell population	2	0.8681	F (2, 25) = 2.424	0.1091	NS
EIFAK2	Interaction	4	2.487	F (4, 27) = 31.39	<0.0001	****
	Infection status	2	2.085	F (2, 27) = 26.32	<0.0001	****
	Cell population	2	1.452	F (2, 27) = 18.33	<0.0001	****
IL-1β	Interaction	4	12.51	F (4, 25) = 7.490	0.0004	***
	Infection status	2	43.35	F (2, 25) = 25.96	<0.0001	****
	Cell population	2	16.87	F (2, 25) = 10.10	0.0006	***
PD-L1	Interaction	4	0.7738	F (4, 27) = 1.708	0.1774	NS
	Infection status	2	11.94	F (2, 27) = 26.36	<0.0001	****
	Cell population	2	3.117	F (2, 27) = 6.880	0.0038	**
PD-L2	Interaction	4	0.4222	F (4, 27) = 2.023	0.1194	NS
	Infection status	2	2.895	F (2, 27) = 13.87	<0.0001	****
	Cell population	2	1.341	F (2, 27) = 6.427	0.0052	**

Transcripts RSAD2, TNF-α and IL-6 had no expression in at least half the replicates for at least one condition, precluding statistical analyses. **Abbreviations:** ANOVA = analysis of variance, CI = confidence interval, df = degrees of freedom, EIFAK2 = eukaryotic translation initiation factor 2-alpha kinase 2, F = F statistic, IFN = interferon, IL = interleukin, NS = not significant, PD-L = programmed death-ligand, RSAD2 = radical S-adenosyl methionine domain-containing 2, t = t statistic, TNF-α = tumor necrosis factor-alpha.

Table S2. Results of statistical analyses by Tukey's multiple comparisons test for Figure 7. Transcript expression of 3 primary human Müller cell isolates was calculated by two-way ANOVA, where data were complete.

**Transcript**	**Müller cell**	**Tukey's multiple comparisons test**	**Mean Difference**	**95% CI of difference**	**Adjusted P-value**	
IFN-β	Isolate 1	Control vs. Mon601	-2.213	-3.083 to -1.343	< 0.0001	****
		Control vs. EHI0578Y05	0.0000225	-0.8699 to 0.8699	> 0.9999	NS
		Mon601 vs. EHI0578Y05	2.213	1.343 to 3.083	< 0.0001	****
	Isolate 2	Control vs. Mon601	-6.279	-7.149 to -5.409	< 0.0001	****
		Control vs. EHI0578Y05	-0.7311	-1.601 to 0.1388	0.1121	NS
		Mon601 vs. EHI0578Y05	5.548	4.678 to 6.417	< 0.0001	****
	Isolate 3	Control vs. Mon601	-2.464	-3.334 to -1.594	< 0.0001	****
		Control vs. EHI0578Y05	-0.04949	-0.9194 to 0.8204	0.9891	NS
		Mon601 vs. EHI0578Y05	2.414	1.544 to 3.284	< 0.0001	****
IFN-α	Isolate 1	Control vs. Mon601	0.2319	-0.8222 to 1.286	0.8484	NS
		Control vs. EHI0578Y05	0.08615	-0.9679 to 1.140	0.9774	NS
		Mon601 vs. EHI0578Y05	-0.1458	-1.200 to 0.9083	0.9368	NS
	Isolate 2	Control vs. Mon601	-2.121	-3.175 to -1.067	0.0001	***
		Control vs. EHI0578Y05	-0.1277	-1.266 to 1.011	0.9579	NS
		Mon601 vs. EHI0578Y05	1.993	0.8549 to 3.132	0.0006	***
	Isolate 3	Control vs. Mon601	-1.102	-2.240 to 0.03676	0.0592	NS
		Control vs. EHI0578Y05	0.1449	-0.9936 to 1.283	0.9462	NS
		Mon601 vs. EHI0578Y05	1.247	0.1926 to 2.301	0.0182	*
EIFAK2	Isolate 1	Control vs. Mon601	-1.441	-1.934 to -0.9474	< 0.0001	****
		Control vs. EHI0578Y05	0.04673	-0.4467 to 0.5402	0.9701	NS
		Mon601 vs. EHI0578Y05	1.488	0.9941 to 1.981	< 0.0001	****
	Isolate 2	Control vs. Mon601	-0.04375	-0.5372 to 0.4497	0.9737	NS
		Control vs. EHI0578Y05	-1.546	-2.039 to -1.052	< 0.0001	****
		Mon601 vs. EHI0578Y05	-1.502	-1.995 to -1.008	< 0.0001	****
	Isolate 3	Control vs. Mon601	-0.124	-0.6175 to 0.3694	0.8087	NS
		Control vs. EHI0578Y05	-0.9641	-1.458 to -0.4707	0.0001	***
		Mon601 vs. EHI0578Y05	-0.8401	-1.334 to -0.3466	0.0007	***
IL-1β	Isolate 1	Control vs. Mon601	-0.8771	-3.335 to 1.581	0.6523	NS
		Control vs. EHI0578Y05	-0.007699	-2.466 to 2.451	> 0.9999	NS
		Mon601 vs. EHI0578Y05	0.8694	-1.406 to 3.145	0.6135	NS
	Isolate 2	Control vs. Mon601	-7.01	-9.286 to -4.734	< 0.0001	****
		Control vs. EHI0578Y05	-0.2814	-2.740 to 2.177	0.9562	NS
		Mon601 vs. EHI0578Y05	6.729	4.271 to 9.187	< 0.0001	****
	Isolate 3	Control vs. Mon601	-2.33	-4.606 to -0.05372	0.0441	*
		Control vs. EHI0578Y05	-0.03724	-2.313 to 2.239	0.9991	NS

Transcript	Müller cell	Tukey's multiple comparisons test	Mean Difference	95% CI of difference	Adjusted P-value	
		Mon601 vs. EHI0578Y05	2.292	0.01649 to 4.568	0.0481	*
PD-L1	Isolate 1	Control vs. Mon601	-1.537	-2.717 to -0.3569	0.0088	**
		Control vs. EHI0578Y05	-0.00495	-1.185 to 1.175	> 0.9999	NS
		Mon601 vs. EHI0578Y05	1.532	0.3519 to 2.712	0.0091	**
	Isolate 2	Control vs. Mon601	-2.78	-3.960 to -1.599	< 0.0001	****
		Control vs. EHI0578Y05	-1.438	-2.618 to -0.2578	0.0146	*
		Mon601 vs. EHI0578Y05	1.342	0.1616 to 2.522	0.0235	*
	Isolate 3	Control vs. Mon601	-1.486	-2.666 to -0.3061	0.0114	*
		Control vs. EHI0578Y05	-0.1865	-1.367 to 0.9936	0.9191	NS
		Mon601 vs. EHI0578Y05	1.3	0.1196 to 2.480	0.0287	*
PD-L2	Isolate 1	Control vs. Mon601	-0.6998	-1.501 to 0.1011	0.0955	NS
		Control vs. EHI0578Y05	-0.01936	-0.8202 to 0.7815	0.998	NS
		Mon601 vs. EHI0578Y05	0.6804	-0.1204 to 1.481	0.1073	NS
	Isolate 2	Control vs. Mon601	-1.25	-2.051 to -0.4489	0.0018	**
		Control vs. EHI0578Y05	-1.312	-2.113 to -0.5109	0.0011	**
		Mon601 vs. EHI0578Y05	-0.06204	-0.8629 to 0.7388	0.9799	NS
	Isolate 3	Control vs. Mon601	-0.9441	-1.745 to -0.1432	0.0184	*
		Control vs. EHI0578Y05	-0.5986	-1.399 to 0.2023	0.1719	NS
		Mon601 vs. EHI0578Y05	0.3455	-0.4553 to 1.146	0.5406	NS

Transcripts RSAD2, TNF-α and IL-6 had no expression in at least half the replicates for at least one condition, precluding statistical analyses. **Abbreviations**: ANOVA = analysis of variance, CI = confidence interval, df = degrees of freedom, EIFAK2 = eukaryotic translation initiation factor 2-alpha kinase 2, F = F statistic, IFN = interferon, IL = interleukin, NS = not significant, PD-L = programmed death-ligand, t = t statistic.
